# Translocation, genetic structure and homing ability confirm geographic barriers disrupt saltwater crocodile movement and dispersal

**DOI:** 10.1371/journal.pone.0205862

**Published:** 2019-08-28

**Authors:** Yusuke Fukuda, Grahame Webb, Charlie Manolis, Garry Lindner, Sam Banks

**Affiliations:** 1 Department of Environment and Natural Resources, Northern Territory Government, Palmerston, Northern Territory, Australia; 2 Research School of Biology, Australian National University, Acton, Australian Capital Territory, Australia; 3 Wildlife Management International Pty. Limited, Karama, Northern Territory, Australia; 4 Research Institute for the Environment and Livelihoods, College of Engineering, IT and the Environment, Charles Darwin University, Darwin, Northern Territory, Australia; 5 Parks Australia, Australian Government, Jabiru, Northern Territory, Australia; Auburn University, UNITED STATES

## Abstract

Translocated saltwater crocodiles (*Crocodylus porosus*) in the Northern Territory (NT) of Australia often return to their original capture sites, which complicates management interventions aimed at reducing human-crocodile conflict. We examined the spatial events implicated in this homing ability, using ARGOS satellite tracking devices. Five large male *C*. *porosus* (3.03 m to 4.02 m TL) were shifted and released 100–320 km from their capture sites, and 3 additional ones (3.67 m to 4.23 m TL) were released at their site of capture as controls. Translocated crocodiles were more mobile than the controls, and moved at sea in the direction of their original capture site. However, they were unable or unwilling to swim around a geographic structure, Cobourg Peninsula, which prevented homing being achieved in all five cases. Two control crocodiles remained near their capture sites, but one, after the first year, made a 900km journey for six months, before returning to its original capture and release site. Genetic analysis of tissue samples from nests across the NT coast demonstrated significant genetic structure across the coast, and confirmed that Cobourg Peninsula contributes to genetic differentiation among populations along the NT coast. These results provide new insights into *C*. *porosus* movements, which have management significance for the maintenance of public safety.

## Introduction

Saltwater crocodiles *Crocodyus porosus* are the most widely distributed of living crocodilians, extending from southern Asia to northern Australia [1,2]. Throughout this range, wild populations were depleted historically, but are recovering at different rates in different countries [2]. Saltwater crocodiles occupy coastal seas, rivers, and a diversity of saline and freshwater wetlands associated with them. They move between rivers at sea [3,4], and are known to make long-distance sea journeys beyond the coastal fringe [2,5]. The coastal movements of *C*. *porosus* create management problems throughout their range, because *C*. *porosus* are large predators that can prey on people [5–8]. Their arrival in coastal areas inhabited by people requires management interventions. The capture and translocation of “problem” *C*. *porosus* has had mixed success because of their homing ability [9].

Homing following translocation from distant sites has been reported for a range of crocodilian species, but only a few such movements have been reconstructed through use of telemetry techniques (e.g. *Alligator missisippiensis* tracked by radiotelemetry [10,11], *C*. *porosus* by satellites [12,13] and *C*. *niloticus* by radio [14] and by satellites [15]).

When *C*. *porosus* were protected in the Northern Territory (NT) of Australia in 1971, the recovery of the wild population was monitored intensively [16–18], as was the concomitant increase in Human-Crocodile Conflict (HCC) [8,19]. “Problem” crocodile removal from Darwin Harbour is an ongoing management intervention aimed at reducing HCC [20]. The fact that 250–300 *C*. *porosus* per year are removed from the harbour [8], where there is limited local breeding, indicates significant immigration via the coast, assumed to be from rivers to the east, west and north of Darwin, but not excluding immigrants from outside Australia. High mobility and random mixing would favour a genetically homogenous population, but none of these assumptions about movement and genetic homogeny have been tested, despite their importance to HCC management. Here we use satellite tracking and DNA analyses to test two null hypotheses:

1. that movements around the coast of the NT are not confounded by natural barriers; and,
2. that there are no genetically distinct subpopulations of *C*. *porosus* across the NT separated by natural barriers.

Given the high mobility and homing capability of *C*. *porosus* [12,13], satellite tracking focused on translocated individuals, but included some control animals released at their site of capture. The satellite tracking data provided detailed movement information about a small sample of translocated and control individuals. This generated a specific hypothesis about a potential movement barrier (the Cobourg Peninsula), which we tested with a genetic dataset on a larger number of individuals sampled from nests in different locations across the NT coastline.

## Materials and methods

### Study area and context

**Fig 1:**
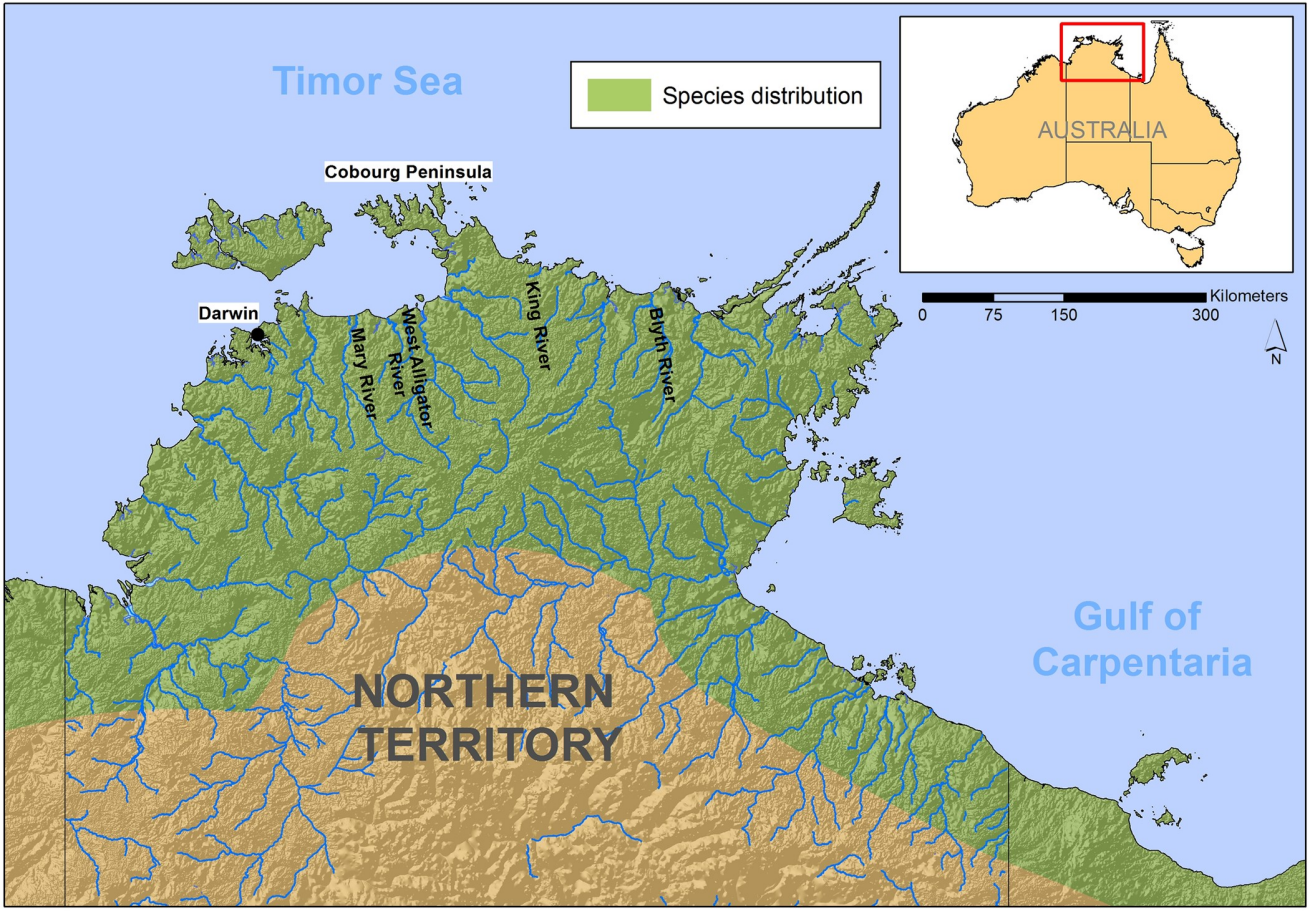
Study area in the Northern Territory of Australia. Distribution of *C*. *porosus* is approximate.

The “Top End” of the Northern Territory (approximately 400,000 km^2^, Fig 1), contains many coastal rivers and creeks, lined with mangroves and under tidal influence near the sea, extending to a diversity of freshwater, non-tidal floodplain wetlands and sand and rock-lime watercourses upstream. All are occupied by *C*. *porosus* at different densities [3,21]. The species roams along the coastline at sea between rivers, and occupies near-coastal islands. The eastern NT coastline is somewhat separated from the western coastline by a land mass associated with Coburg Peninsula (Fig 1).

The Top End climate is the tropical monsoon with a distinct wet season (November to April) and dry season (May to October). Available wetlands expand during the wet season and contract during the dry season [3,22], when many temporary waterbodies dry out if early rains are delayed. Crocodiles tend to move back into permanent water areas during the dry season, but are sometimes forced to aestivate in drying mud. In the NT, there is limited intensive agriculture and most coastal and riparian habitats remain intact [21].

### Ethics

This study was conducted in accordance with the Code of Practice on the Humane Treatment of Wild and Farmed Australian Crocodiles [23]. The protocol was approved by the Animal Ethics Committee of the Australian University (Animal Ethics Protocol Number A2017/11) and the Parks and Wildlife Commission of the Northern Territory (Research Permit Number 7902). It is a contribution to the Northern Territory Government’s crocodile management programs [20,24].

### Satellite tracking

To examine movements of crocodiles relative to the Cobourg Peninsula, we used KiwiSat 101 satellite transmitters with specifications following Read et al. [12]. Transmitters were attached to the nuchal crest of the crocodiles [25] and data were retrieved with the ARGOS system. The results for 8 crocodiles are examined here. All were caught in the wild with traps and skin harpoons [26]. Based on total length (TL), six were mature (3.67 m to 4.23 m TL), one immature (3.03 m), and one possibly mature (3.27 m TL). On the western side of the Cobourg Peninsula, five crocodiles were caught in the Mary River, and either released near their site of capture (N = 3), transported 100 km further west and released at sea (N = 1), or moved 320 km to the eastern side of Cobourg Peninsula and released in the Blyth River (N = 2) (Fig 1). Conversely, crocodiles caught east of Cobourg Peninsula in the Blyth River (N = 1) and King River (N = 1), were translocated west and released in the Mary and West Alligator Rivers, respectively.

We filtered the ARGOS readings, using the R package argosfilter [27]. The AROGS locations filter reduces noises in ARGOS data, based on ARGOS Location Class, maximum swimming speed of a tracked animal (threshold 2 meters per second), distance between successive locations, and turning angles (spikes with angles smaller than 15 and 25 degrees with extension higher than 2500 m and 5000 m to be removed) [28]. We sorted the extracted ARGOS data in chronological order based on the date and time of the signal reception, and mapped them for each of the tracked crocodile using ArcGIS.

### Genetic analysis

**Fig 2:**
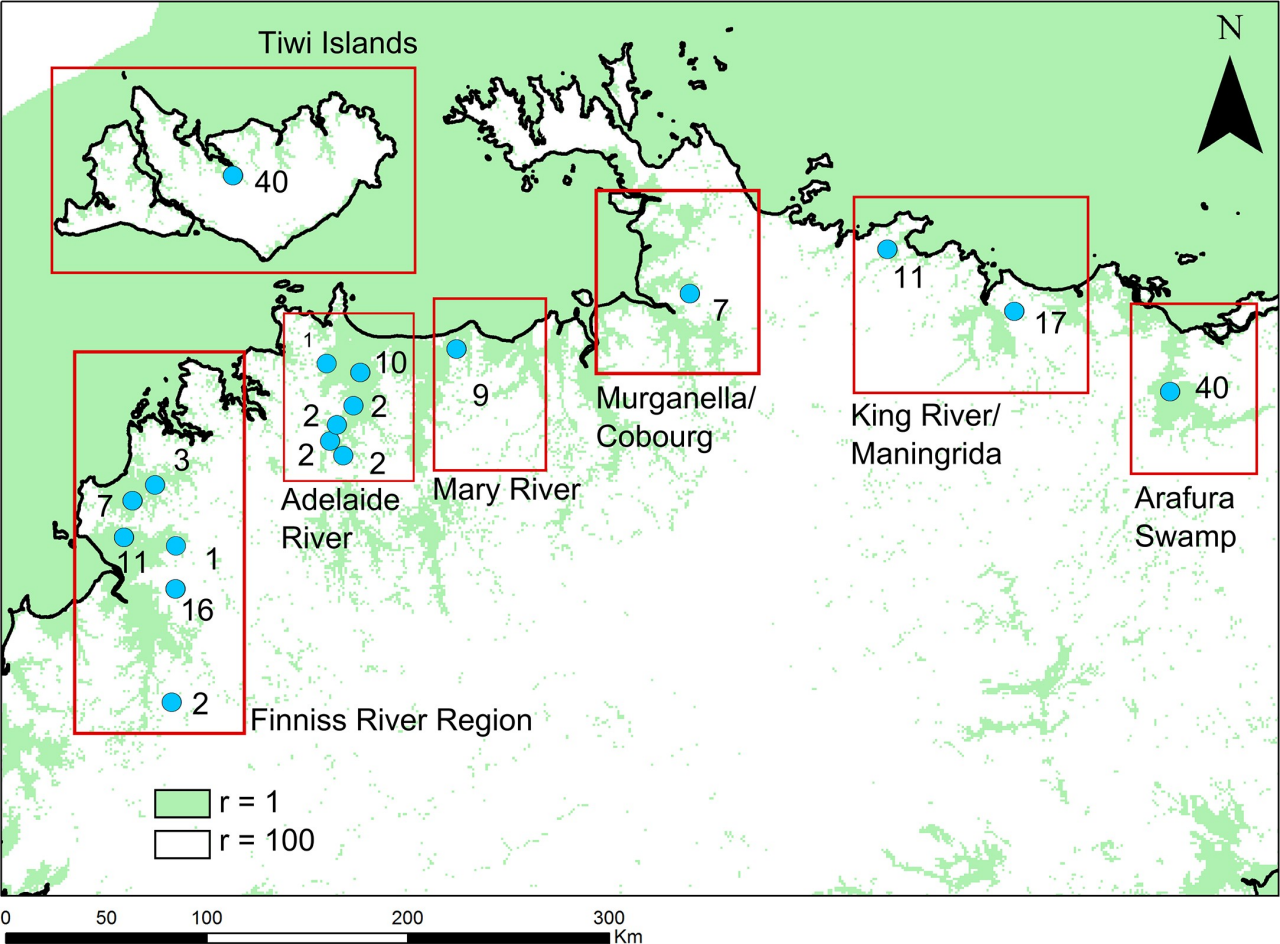
Nest locations (blue dots) and skin samples from each nest (numbers) used in the genetic analysis. Red boxes indicate site groupings used for population genetic diversity analysis. Note that the samples collected at the Cobourg Peninsula (purple) were used for the genetic assignment analysis only. Green indicates actual and potential crocodile habitat during the wet season, coded as resistance = 1 in the analyses, and white areas of dry land, coded as resistance = 100 (see text).

We collected 192 *C*. *porosus* tissue samples from 19 locations across the Top End (Fig 2). All were pieces of skin from hatchlings that failed to develop to term during incubation. Eggs were collected at different locations as part of the NT’s commercial egg-ranching program [20,29].

DNA extraction and genotyping was conducted at Diversity Arrays Technology (Canberra) using the DArTseq approach [30]. DArTseq uses genome complexity reduction and NGS approaches conceptually similar to RADseq, in this case based around a *SphI* enzyme digestion of genomic DNA and Illumina HiSeq 2500 sequencing. Initial sequence processing and SNP-calling by DArT yielded 11,497 single-nucleotide polymorphism (SNP) loci. We used the *dartR* package in R 3.4.0 [31] to filter SNPs according to call rate (CallRate > 0.95), repeatability (RepAvg > 0.95) and dropping ‘secondary’ SNPs (ranked by polymorphic information content) within sequences with more than one SNP present. We then examined a histogram of the F_IS_ values (inbreeding coefficient) at the filtered SNP loci (S1 Fig). After this exploratory analysis, we did not filter our SNPs further. As an exploratory population genetics analysis, we estimated observed and expected heterozygosities within the major sampled regions (Fig 2) in DartR, as well as by pairwise F_ST_ using StAMPP [32] with 100 bootstraps.

To investigate whether the Cobourg Peninsula acts as a barrier to mixing of subpopulations, we fitted linear mixed-effects models to pairwise genetic similarity data among individuals, to compare models featuring fixed effects representing least-cost distance along watercourses and a putative additional barrier corresponding to the distinction between populations on either side of the Cobourg Peninsula. The genetic similarity metric that we used was a simple Identity-by-State (IBS) measure calculated in the R package SNPrelate [33] on the filtered set of allele frequencies, imposing an extra minor allele frequency filter (retaining SNPs with MAF > 0.05) with linkage R^2^ threshold criterion of 0.1, yielding 5,675 SNPs). Because multiple individuals came from most nests sampled (Fig 2), our data points were the average pairwise IBS values among individuals sampled within the 19 sampling locations.

The basic ecological distance estimates among sampling locations were least-cost distances calculated in the R package gdistance [34], using a landscape resistance surface with a resistance value of 1 for marine and freshwater areas corresponding to the northern Australian wet season. Areas unsuitable for crocodile movement (i.e. dry land areas not classified as suitable wet season habitat) were coded with an arbitrary resistance value of 100, indicating 100+ times as difficult to move through non-habitat than habitat. The effect of selecting these resistance values was that our ecological-distance matrix linked sampled crocodiles by aquatic movement pathways, excluding ‘shortcuts’ across land. We added a second candidate explanatory variable corresponding to binary 1/0 distances representing the putative Cobourg Peninsula barrier. We fitted linear mixed-effects models in the *nlme* R package [35] using the maximum likelihood population effects (MLPE) correlation structure to account for multiple pairwise comparisons among a set of populations. We constructed the MLPE correlation structure using the R script available at https://github.com/nspope/corMLPE_unsupported/blob/master/corMLPE_unsupported.R and provide our own R scripts to analyze our genetic data (S1 File). We fitted models featuring effects of: (1) ecological distance and the putative Cobourg Peninsula barrier; (2) ecological distance alone; (3) the putative Cobourg Peninsula barrier alone; and, (4) the intercept alone. We compared models with the AIC and evaluated significance of our candidate exploratory variables within each model.

As a supporting analysis, we used a genetic assignment test approach to estimate the ancestry of each sampled individual in a set of pre-defined geographic regions, using the R package *assignPOP* [36]. Given our samples were from harvested eggs, our interest was in detecting recent ancestry from outside the sampling region. We re-defined the Darwin/Kakadu region by combining the Adelaide River, Mary River and Murganella Creek, and the northern Arnhem Land region by combining the King River/Maningrida and Arafura Swamp sampling areas. We kept the regions of the Tiwi Islands and Finniss River unchanged as these were distinct from the adjunct regions. We applied the MAF <0.05 filtering and used the *assign*.*kfold* function of the *assignPOP* package to estimate ancestry coefficients of each individual in each of the geographic ‘reference populations’. For the analysis, we used the 50% of SNPs with the highest F_ST_ for assignment. We iteratively removed 10% of individuals from each population for assignment and used the remaining 90% of individuals for the reference assignment population, until all individuals had been analysed using the support vector machine (SVM) classification model [36].

## Results

### Satellite tracking

**Fig 3:**
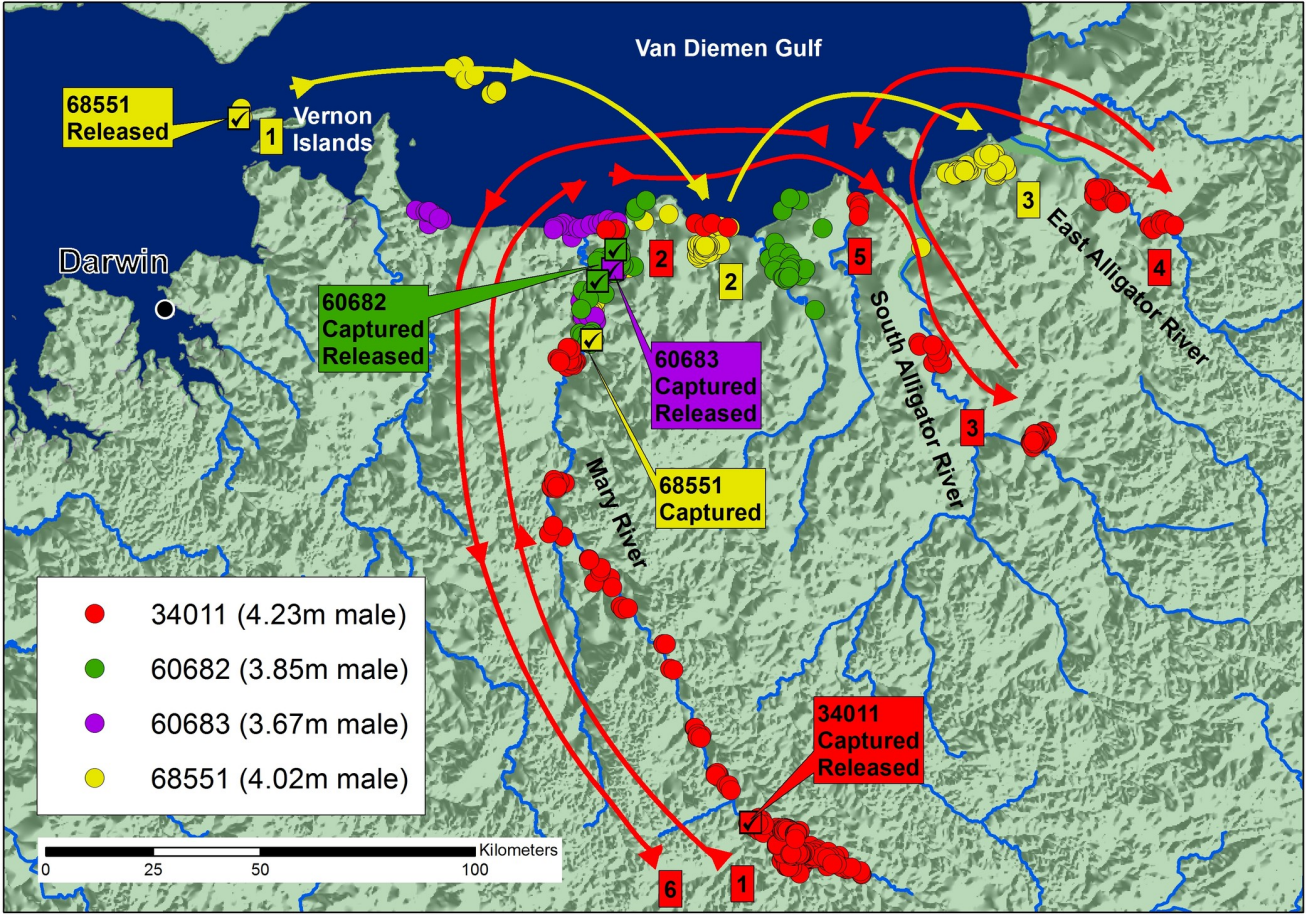
Locations and likely paths of movement of four crocodiles caught in the Mary River, three of which were released near the site of capture (60682, 60683, 34011) and one (68551) translocated 100km west and released at sea.

The tracking results of the four animals caught and released on the western side of the Cobourg Peninsula, are summarized on Fig 3. Crocodiles 60682 (3.85 m TL; 268 days of tracking) and 60683 (3.67 m TL; 48 days of tracking) remained in and around their capture site, and when they did venture to the coast, they stayed within 15 km of the mouth of the Mary River and showed no strong directional movement. Crocodile 34011 (4.23 m TL; 930 days of tracking) was caught 170 km upstream in the Mary River. During its first year, it remained at the capture and release site, but in October 2009, following heavy rains, it travelled downstream to the coast, then journeyed east around 120 km to the East Alligator River, before returning to the Mary River and travelling back upstream to its capture site. The journey involved some 900 km in six months. Crocodile 68551 (4.02 m TL; tracked 180 days) was caught in the downstream Mary River, translocated 100 km further west and released at sea near the Vernon Islands. Within 25 days, it had returned to its capture site in the Mary River, but it then travelled back out to the coast and eastward, moving between two specific inland coastal wetlands, 25 km and 90 km east of the Mary River. Details of each individual’s movement are provided as Supporting Information (S2 File).

The results are consistent with the view that the crocodiles on the western side of the Cobourg Peninsula have one or more activity centers within and between adjoining rivers, and their ability to move east or west, or upstream (south) and downstream (north), between them. Some form of refined navigational ability exists, which reflects itself in the rapid homing of the translocated individual. None of the crocodiles followed the coastline beyond the East Alligator River up towards Cobourg Peninsula.

**Fig 4:**
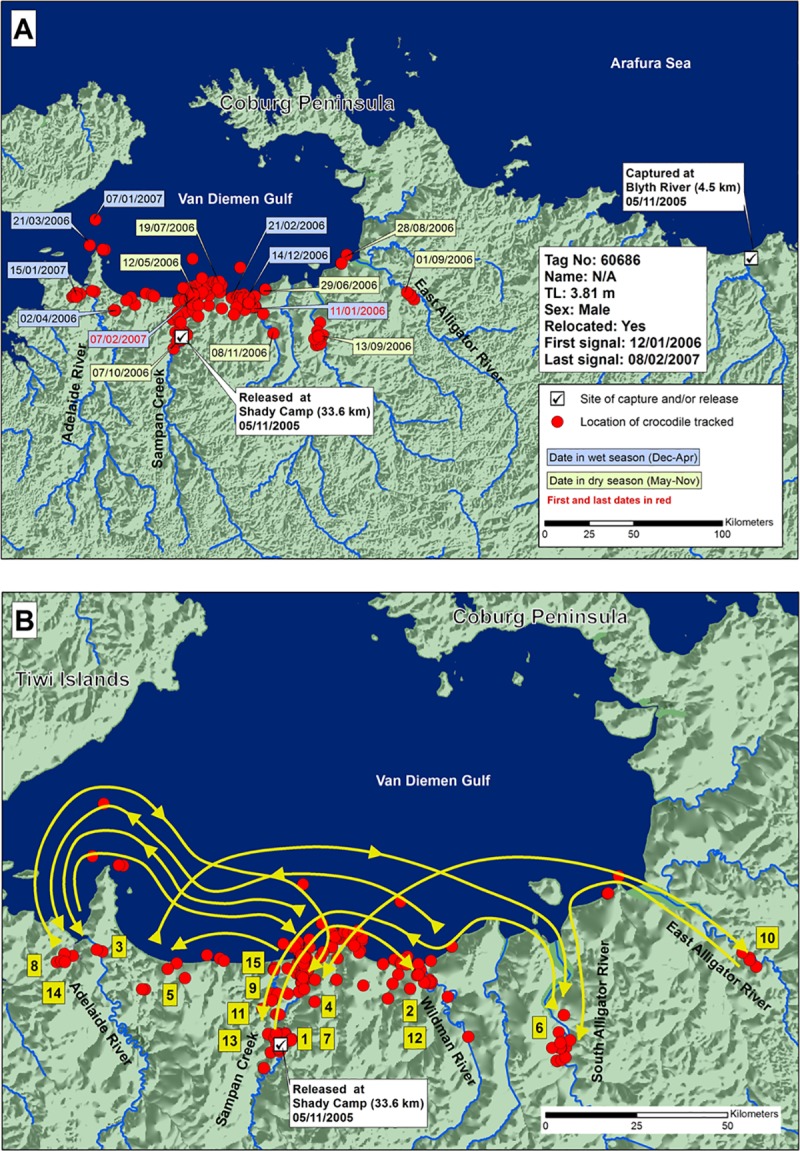
A) Locations and dates for crocodile 60686 translocated from the Blyth River east of Cobourg Peninsula to the Mary River, and B) likely paths chronologically numbered of crocodile travel.

In contrast to these results, the crocodiles translocated between the western and eastern sides of Cobourg Peninsula showed different mobility patterns. Crocodile 60686 (3.81 m TL; 396 days tracking) was translocated from the Blyth River on the eastern side of Cobourg Peninsula, 320 km west to the Mary River (Fig 4). It was highly mobile around the release site, moving back and forth along the coast from the Adelaide River to the East Alligator River (160 km), and entering Sampan Creek in the Mary River and the South Alligator River. The closest point to the original capture site it reached, upstream in the East Alligator River, was approximately 200 km from the Blyth River capture site, but separated from it by land. No seasonal trend in movement was detected, although the tracking period encompassed complete wet and dry seasons. By the last transmission, the crocodile had returned to the release site in the Mary River.

**Fig 5:**
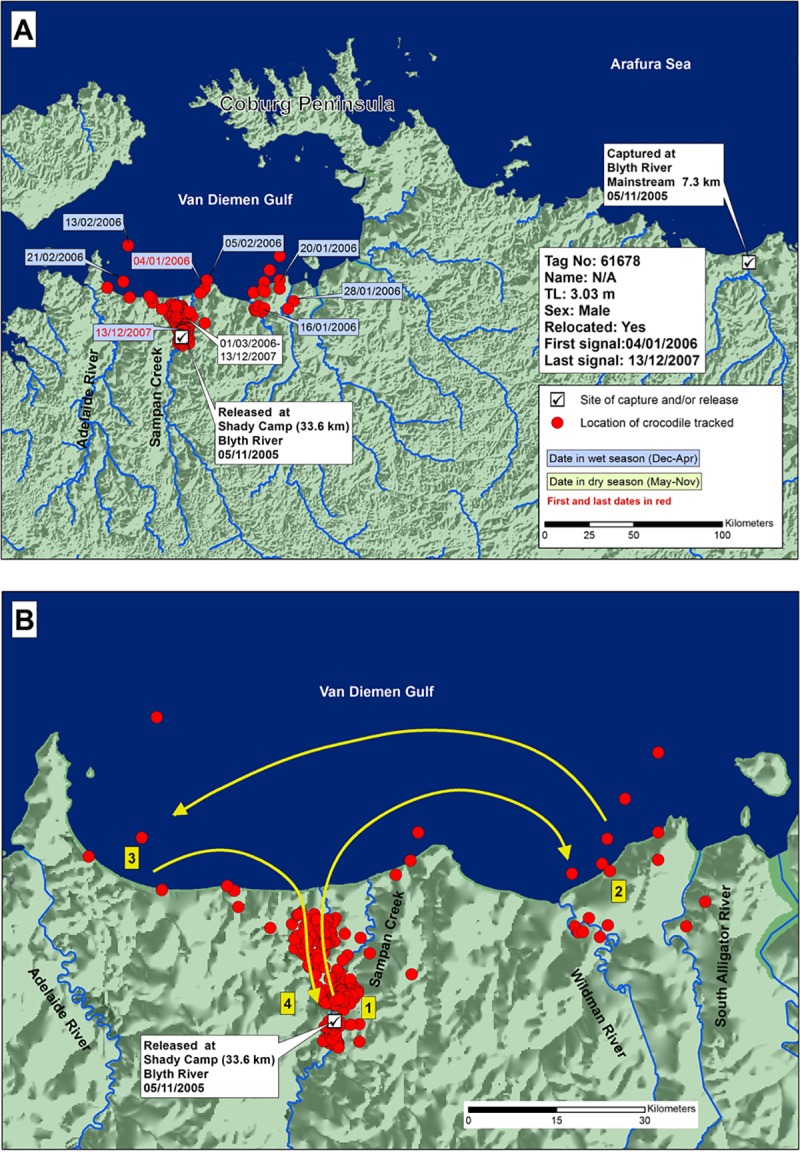
A) Locations and dates for translocated crocodile 61678, and B) likely paths chronologically numbered of crocodile travel.

Crocodile 61678 (3.03 m TL; tracked 708 days) was the smallest male in the study, and was assumed to be immature. It also was caught in the Blyth River and released 320 km to the west in the Mary River (Fig 5). It roamed east and west along 100 km of coastline in the first two months after release, then returned and stayed in a single area near the release site in Sampan Creek, Mary River. Almost two complete cycles of wet and dry seasons were included, with no seasonal movements obvious.

**Fig 6:**
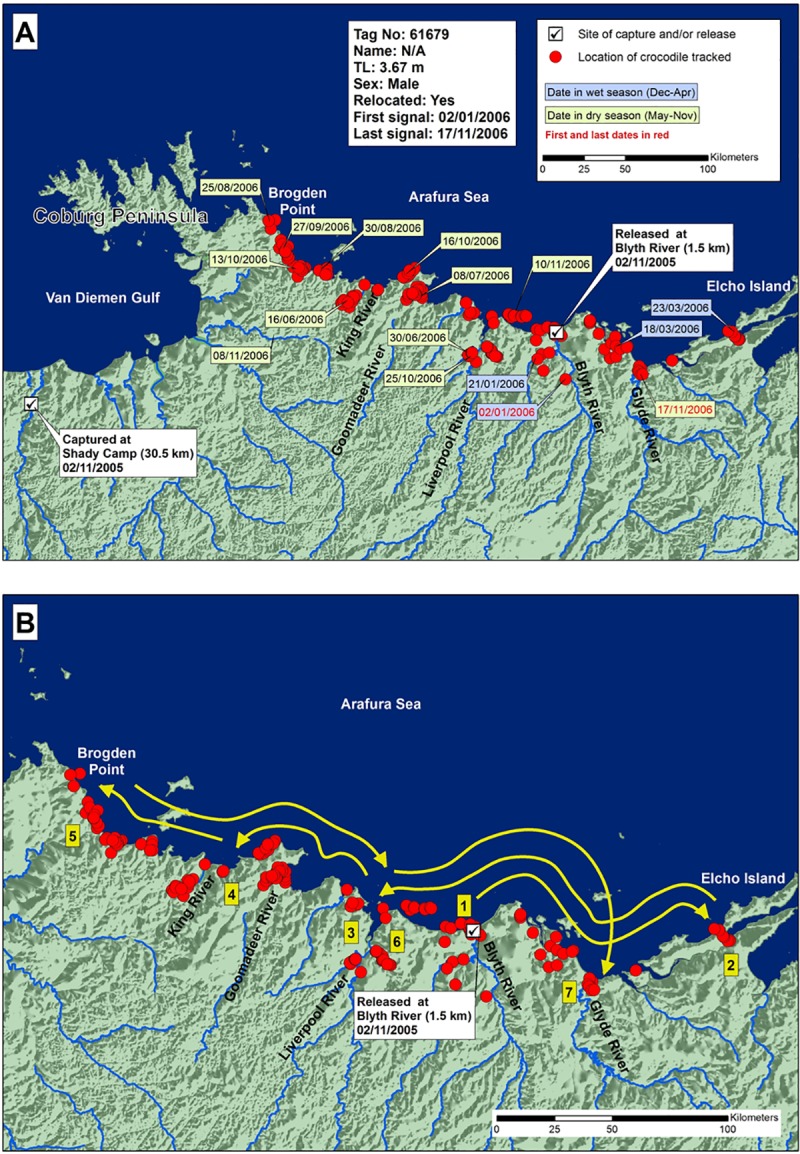
A) Locations and dates for translocated crocodile 61679, and B) likely paths chronologically numbered of crocodile travel.

Crocodile 61679 (3.67 m TL; tracked 316 days) was translocated from west to east of Cobourg Peninsula. It was captured in the downstream Mary River, and translocated and released in the Blyth River (Fig 6), 320 km east. In the 316 days of tracking, it roamed back and forth over 300 km of coastline between Elcho Island in the east and Brogden Point in the west, entering every major river in-between, including the Blyth, Glyde, Goomadeer and King, and Liverpool Rivers, without going far upstream in any of them. The crocodile continued to move until transmissions ceased at the mouth of the Glyde River. There was no apparent seasonal pattern in the movements. It did not move out onto Cobourg Peninsula when it reached the full extent of its westward journey, but rather returned to the east.

**Fig 7:**
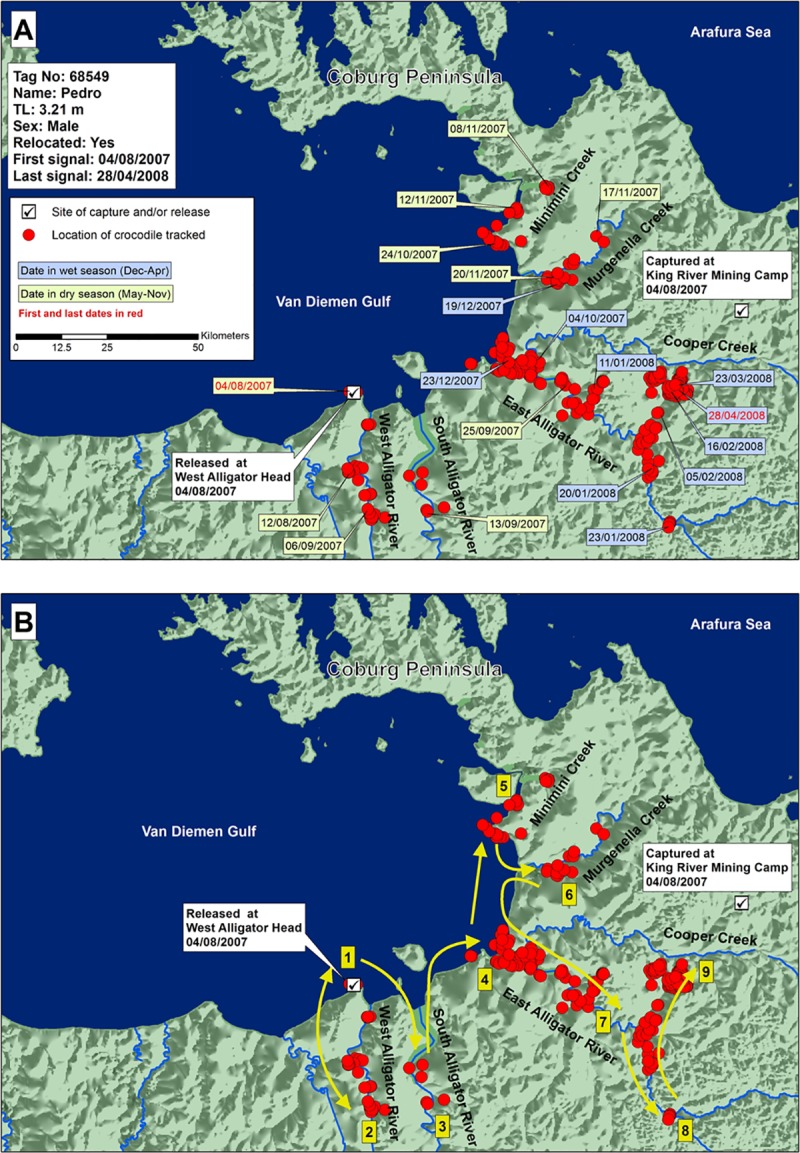
A) Locations and dates for translocated crocodile 68549, and B) likely paths chronologically numbered of crocodile travel.

Crocodile 68549 (3.21 m TL; perhaps still immature; tracked 268 days) was captured upstream in the King River to the east of Cobourg Peninsula, and released near the West Alligator River mouth (West Alligator Head, Fig 7), west of Cobourg Peninsula. It moved east, in the direction of its capture site, backtracking when it started to head north. It entered all major rivers between the West Alligator River and Minimini Creek. After visiting the West Alligator River, South Alligator River, Minimini Creek, and Murgenella Creek, the crocodile travelled upstream in the East Alligator River, to a point where it began heading away from its capture site. It then backtracked and moved out onto a floodplain, to a position 90 km from its release site and 25 km from its capture site, but separated from it by dry land.

The four crocodiles translocated demonstrated an apparent navigational ability, consistent with homing, but none followed the coastline out along the Cobourg Peninsula.

### Genetic analysis

**Fig 8:**
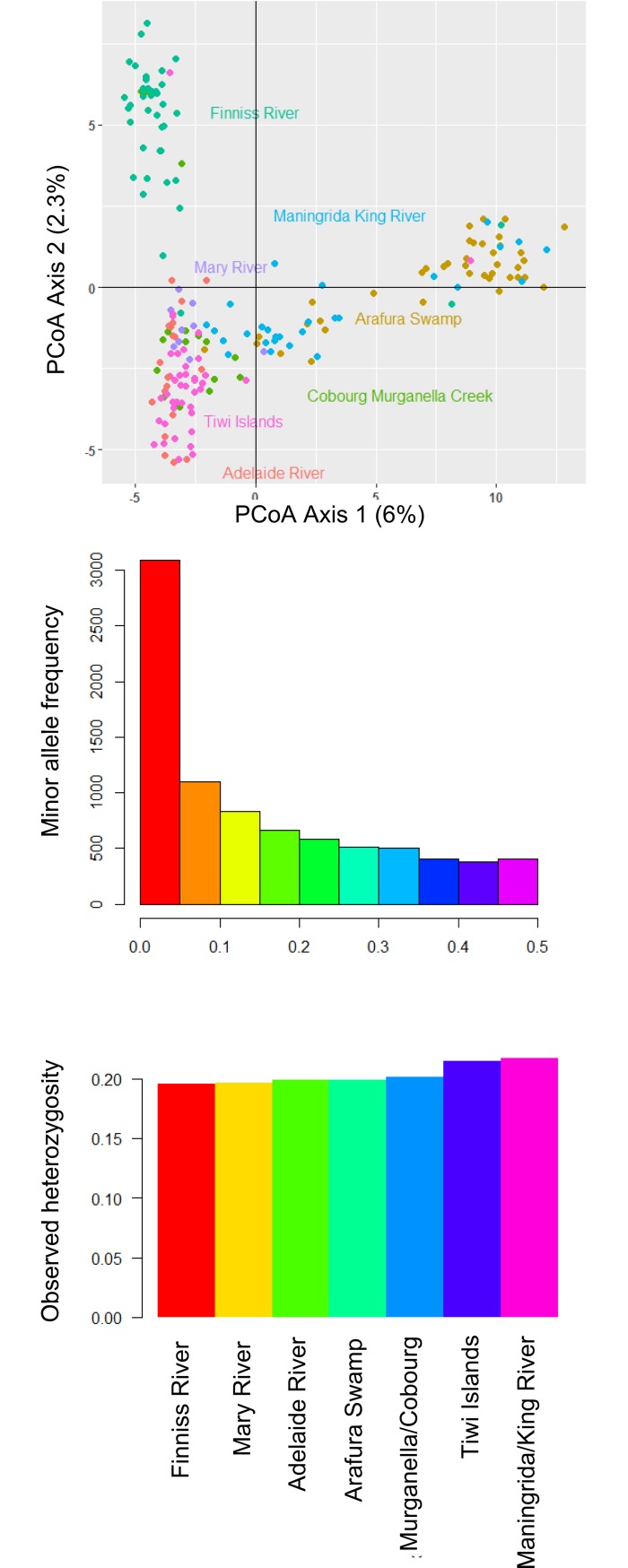
Principal coordinates analysis of individual SNP genotypes, minor allele frequencies across post-filtering SNP loci and observed heterozygosities in each region.

Basic genetic diversity metrics from the 8,312 SNPs that were retained post-filtering, including the distribution of minor allele frequencies and observed heterozygosities within sampling regions (average H_o_ = 0.203), are shown in Fig 8. A PCoA ordination of individual genotypes (Fig 8) shows that individuals were broadly grouped by sampling region. Relative to the Cobourg Peninsula, the first PCoA axis separated the eastern regions (Maningrida, King River, Arafura Swamp) from the western regions, while the second PCoA axis was largely a north-south divide on the west, separating the Finniss River samples from those between Darwin and the Cobourg Peninsula. The Tiwi Islands samples clustered closely with those from the nearby Adelaide and Mary Rivers. Mean pairwise F_ST_ among regions was 0.037, with all pairwise values being significant (Table 1).

**Table 1:** F_ST_ estimates between saltwater crocodile sampling regions. Pairwise F_ST_ estimates in bold represent sampling locations separated by the Cobourg Peninsula. See S1 Table for bootstrap F_ST_ estimates with 95% confidence intervals, none of which crossed zero.

	Cobourg Murganella Creek	Adelaide River	Finniss River	Mary River	Arafura Swamp	Maningrida King River
Adelaide River	0.019					
Finniss River	0.023	0.038				
Mary River	0.018	0.029	0.037			
**Arafura Swamp**	**0.062**	**0.076**	**0.075**	**0.069**		
**Maningrida King River**	**0.027**	**0.041**	**0.044**	**0.034**	**0.015**	
Tiwi Islands	0.013	0.023	0.032	0.023	**0.061**	**0.031**

**Fig 9:**
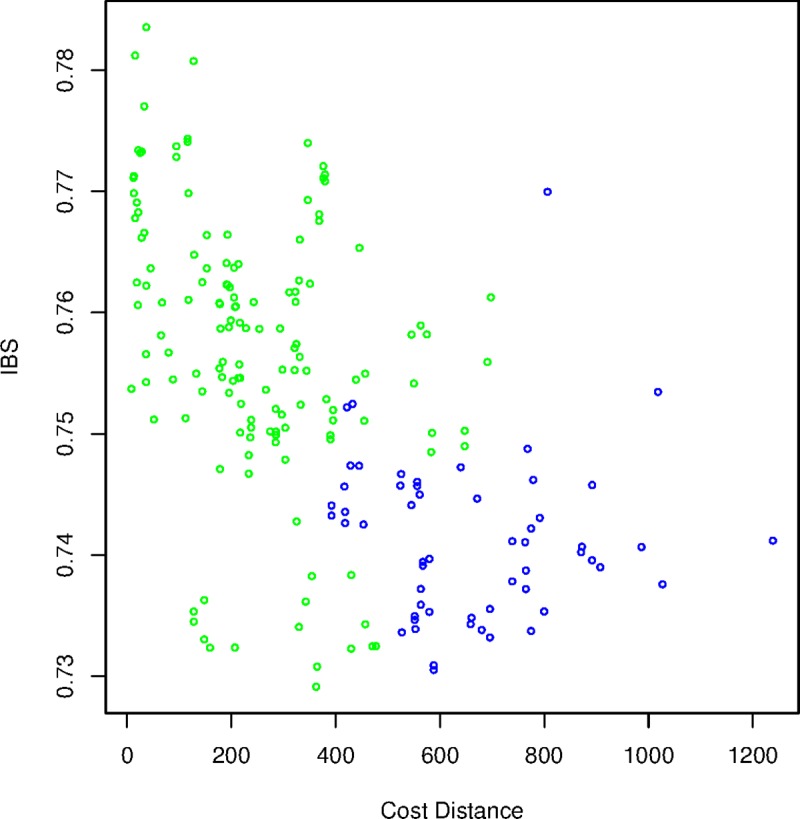
Scatterplot of mean Identity-by-State (IBS) among individuals in each of the 19 sampling locations in relation to ecological cost distance between each point. Pairwise comparisons spanning the Cobourg Peninsula are colour-coded blue and those on the same side of the peninsula are coded green.

After MAF filtering, we used 5,291 loci to calculate IBS among samples. The scatterplot of IBS and ecological distance (Fig 9) with a narrow range of ecological distance values (~400–750 km) allowed comparison of samples from east and west of Cobourg Peninsula. All pairwise distances less than 400 km were among sampling sites on the same side of the Cobourg Peninsula and all pairwise distances greater than 750 km involved across-Cobourg comparisons. We fitted models to the full dataset and then repeated them with distances restricted to this geographic window. For the full dataset, the ecological distance-only model was highest-ranked, and featured a significant decline in IBS with increasing ecological distance among crocodiles (Table 2). The ecological distance and Cobourg barrier model was second-ranked and featured a significant effect of ecological distance, but not the peninsular barrier. We considered the more appropriate models to be those fitted to the restricted dataset, as the two candidate explanatory variables were highly correlated (-0.734) across the full dataset but only weakly correlated (-0.11) in the distance-restricted dataset. Essentially, the distance and putative barrier variables were confounded outside of the restricted dataset. Of these models, the model featuring an effect of the putative peninsular barrier was best supported according to AIC (Table 2) and featured a significant negative effect of the putative barrier on IBS. Ecological distance was not a significant explanatory variable in any of the models fitted to the reduced dataset (Table 2).

**Table 2:** Comparison of linear mixed-effects models with maximum likelihood population effects (MLPE) correlation structure to investigate relationships between mean Identity-by-State (IBS) in saltwater crocodile genotypes among locations, ecological cost distance, and the effect of a putative barrier to gene flow at the Cobourg Peninsula.

Model		Estimate	SE	t	P	AIC	ΔAIC	MLPE Rho
**Full dataset**								
**1**	**Intercept**	0.762	0.002	316.161	0	-1300.232	11.655	0.199
	**Ecol distance**	-0.0003	3.13E-06	-5.54	0			
	**Cobourg**	-0.002	0.002	-0.696	0.487			
**2**	**Intercept**	0.763	2.38E-03	321.222	0	-1311.887	0	0.205
	**Ecol distance**	-0.0003	3.17E-06	-8.923	0			
**3**	**Intercept**	0.756	2.04E-03	369.939	0	-1297.136	14.751	0.173
	**Cobourg**	-0.012	1.85E-03	-6.7073	0			
**4**	**Intercept**	0.753	2.40E-03	314.346	0	-1270.18	41.707	0.198
**Reduced dataset**						
**1**	**Intercept**	0.751	0.004	189.614	0	-357.435	24.046	0.337
	**Ecol distance**	0	0	-0.659	0.513			
	**Cobourg**	-0.009	0.002	-4.239	0			
**2**	**Intercept**	0.75	0.005	152.735	0	-355.809	25.672	0.313
	**Ecol distance**	0	0	-1.377	0.174			
**3**	**Intercept**	0.749	0.002	312.816	0	-381.481	0	0.335
	**Cobourg**	-0.009	0.002	-4.35	0			
**4**	**Intercept**	0.745	0.002	304.914	0	-377.738	3.743	0.322

**Fig 10:**
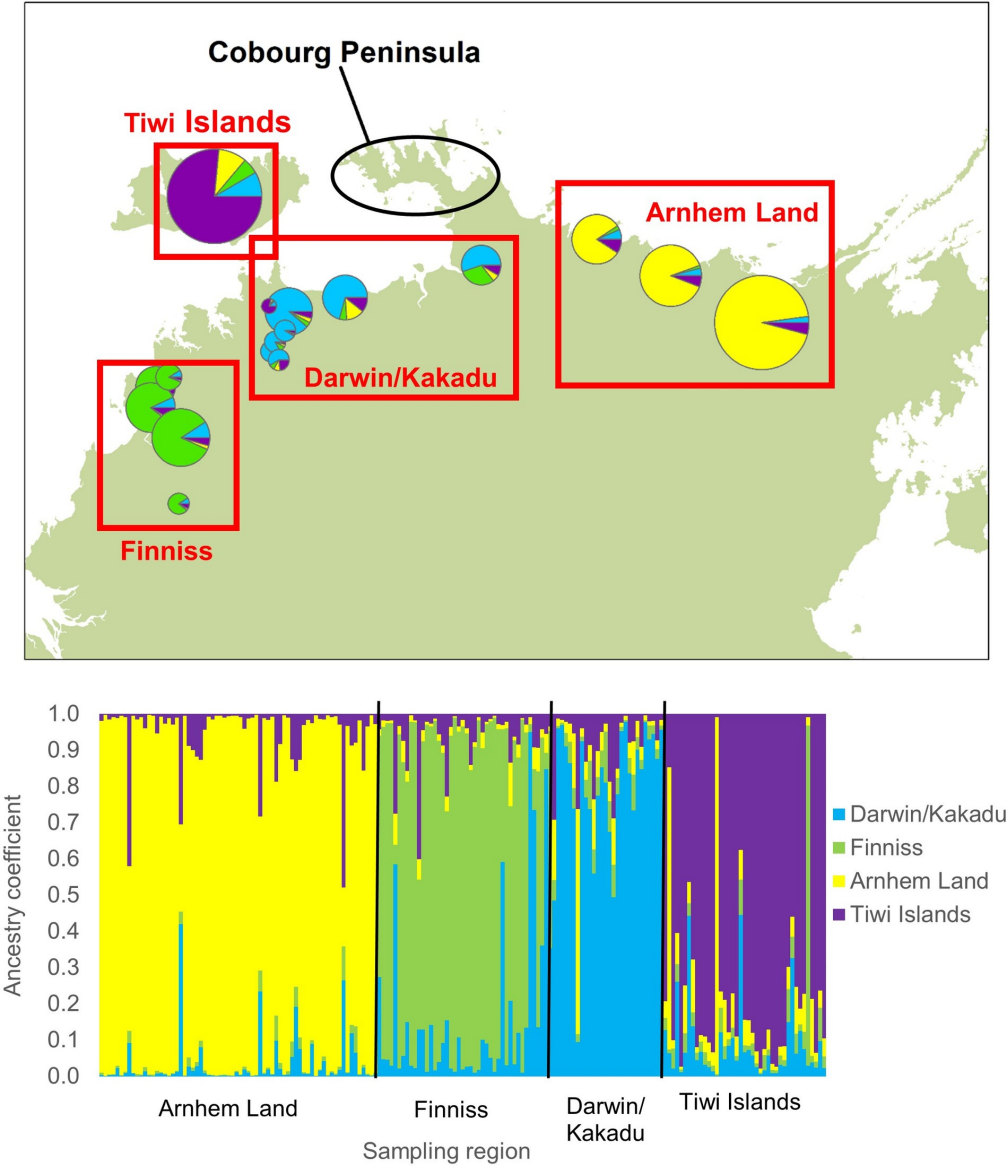
Genetic assignment of each individual to the pre-defined regions of the Arnhem Land, Darwin/Kakadu, Finniss River, and Tiwi Islands. The size of pie-charts is relative to the sample size of each population as shown in Fig 2. The calculated ancestry coefficients for each individual are presented in S3 File.

The genetic assignment analysis showed relatively clear separation among the sampled regions, especially between those in the west of the Cobourg Peninsula and Arnhem Land (Fig 10). Of the samples collected from the northern Arnhem Land region, only 3% of individuals had an ancestry probability of greater than 0.2 in the sampled regions west of the Cobourg Peninsula (S3 File). Of the samples collected in the three grouped regions west of the Cobourg Peninsula, only 6% of individuals had an ancestry probability of greater than 0.2 in the northern Arnhem Land region east of the Cobourg Peninsula. This contrasted with 17% of individuals in the regions west of the Cobourg Peninsula having ancestry probabilities greater than 0.2 in neighbouring regions on the same side of the Cobourg Peninsula. These data indicate some gene flow occurs around the Cobourg Peninsula but suggests that it occurs less commonly than gene flow among neighbouring populations either side of this coastal feature.

## Discussion

Translocated *C*. *porosus* have a high probability of returning to their site of capture [9], but few results demonstrate how this is achieved except for those from Queensland, Australia [12,13]. The 4.02 m crocodile (68551) on the western side of Cobourg that we released 100 km west of its capture site returned within 25 days (Fig 3). This result parallels to that of a previous study in which 3.1-m and 3.8-m males captured on the eastern and western sides of Cape York Peninsula in Queensland, respectively, and released 56 km and 99 km away on the same side, returned to their capture sites within a month [12].

However, unlike the NT crocodiles we studied, a 4.5-m *C*. *porosus* caught on the western side of Cape York Peninsula, and released on the eastern side, stayed near the release site for three months then returned 411 km to its capture site within a month, possibly exploiting local surface currents around Cape York [12,13]. Cape York was clearly not an effective barrier to such homing movements, at least for large individuals. In contrast, Cobourg Peninsula was not crossed by any of the five translocated crocodiles in our study, nor by the three control crocodiles released at the capture site.

How Cobourg Peninsula interferes with east-west and west-east movements of *C*. *porosus* is unknown. Ocean currents could be involved as in Queensland [13], but the coastal oceanography around Cobourg Peninsula is complex, and not particularly well understood. There are some significant deep channels on the eastern side (<164 m), and large tides (<8 m) [37] combined with seasonal monsoons, in which wind strength and direction change [38], which may interfere with animal movements, perhaps through the avoidance of rough surface currents flowing against them when travelling at sea. Satellite tracking of false killer whales (*Pseudorca crassidens*) in the Cobourg Peninsula area has shown that they rarely entered the western side of the Cobourg Peninsula, into Van Diemen Gulf, despite occupying shallow (<10 m depth) waters within 10km of the coast, on the eastern side [39], which could also be related to currents.

Given the unidirectional movements associated with homing in the *C*. *porosus* studied here, their ability to follow the shoreline and negotiate Cobourg Peninsula could create a navigational conflict, because they would need to detour by swimming north in parts, away from the direction of their capture location. To return to a location like the Blyth River, east of the Mary or Alligator Rivers (68549, 60686), crocodiles would need to go north, west, north, and then south around Cobourg Peninsula to get back on their eastward trajectory, which may be too sophisticated a navigational challenge for them, although such navigational detours have been observed in pigeons and other animals [40–42].

Crocodiles would also need to leave the highly productive Alligator Rivers region, which supports a large population of crocodiles and abundant nesting [18,21,22], for a northward journey into an area without major rivers, swamps or nesting habitats. The long-distance journey by the large control animal (34011) during the wet season coincided with the breeding season [3,43]. The movement is more consistent with some form of biological need, such as a breeding migration rather than random movements, but indicates a very precise ability to home to its original capture site.

Not all relocated crocodiles sustained movements consistent with homing. The smallest male in this study, caught in the Blyth River and released in the Mary River (61678), settled at its new release site for almost two years. It made only one brief coastal journey, two months after release, travelling 60 km east and then west of the mouth of the Mary River, before returning upstream to the place where it had settled at the release site. This contrast may indicate random differences between individual crocodiles, or differences in the desire to home between immature and mature males, with presumably different histories of individual territoriality at their original capture site. Although *C*. *porosus* shows apparent territoriality at an early age [44,45], territoriality in immature crocodiles may not be as evidently developed as in mature individuals.

Many animals, including migratory birds and marine turtles, sense the earth’s magnetic field to collect navigational information [46–49]. There is anecdotal evidence that crocodilians also use the magnetic field for navigation [50]. Twenty individuals of *C*. *acutus*, *C*. *crocodilus*, and *C*. *moreletii*, in different sizes ranging from 1.4 and 4.0 m, were attached magnets on the head before being translocated (3–120 km) and released in Mexico. Despite the high probability of those without magnets returning to capture sites, none of them returned by July 2008 (the earliest release was in September 2004 and latest in December 2007). Similar observations on *C*. *acutus* using a magnet to disrupt their homing behavior have been reported in popular media in the USA [51]. It has been suggested that crocodilians develop homing ability with a magnetic map to detect their position and orientation in relation to a target [10,15]. They may also use other cues, such as landscape, astronomy or scents, possibly in combination [15], although the celestial and olfactory cues were not requirements for some *A*. *mississipiensis* juveniles to reach home from unfamiliar sites [10]. In our study, it is difficult to identify which cues the translocated crocodiles relied upon.

Combrink [15] documented the homing ability of a relocated 2.7m female crocodile (*C*. *niloticus*) that travelled 178.3 km to its capture site over 136 days. Although the homing track included approximately 18 km of travelling on land, that geographical barrier did not prevent the animal from travelling was not present. Similar overland homing over a much shorter distance by translocated *A*. *mississippiensis* has also been reported [11]. None of our tracked crocodiles, however, showed overland movement between waterbodies. This may be a reflection of the biological differences between the species (e.g. terrestrial locomotion, dehydration tolerance, etc), or a consequence of the small sample size of this study.

None of the eight crocodiles that were tracked, from either east or west of the Cobourg Peninsula, ventured along the Peninsula itself, despite the apparently strong need of some individuals to return to their capture site. This observation is consistent with the peninsula being barrier to dispersal of larger males, for reasons still unknown. If such a barrier exists, there is the potential for genetically distinct subpopulations on either side of the Cobourg Peninsula. Even though the number of the crocodiles tracked by satellites was rather small (N = 8), the genetic analysis that sampled 192 crocodiles across the study area identified greater differentiation across the Cobourg Peninsula than over equivalent distances elsewhere (Fig 9), allowing us to reject the possibility of genetic homegeneity across the NT coastline.

By restricting the spatial genetic analyses to a smaller geographic scale, where we had adequate sample sizes for pairwise genetic comparisons either side of the Peninsula versus the same side of the Peninsula, the results were clear that crocodiles on the same side of the Peninsula were more genetically similar than those across the Peninsula. This result that Cobourg Peninsula is a barrier to the coastal movement of crocodiles is further supported by the assignment analysis that shows lower migrant ancestry across the peninsula compared to among regions on the western side of the peninsula. It should be noted that the one individual that was sampled in the Darwin/Kakadu region but showed high probability of ancestry in the Arnhem Land region also had some ancestry from the Tiwi Islands, suggesting that this crocodile is unlikely to be the first generation of migrants from Arnhem Land.

The approach that we used to analyze the genetic data can estimate the effects of environmental features on relative rates of dispersal [52,53], but does not tell us the absolute migration rates across the Peninsula or elsewhere in the study area. Increased sampling in the immediate vicinity of Cobourg Peninsula could improve our ability to define where and how the barrier operates. Other genetic methods can demonstrate individual dispersal events and rates [53], and it is our intention to apply them to movement and dispersal across northern Australia.

From a management perspective aimed at reducing HCC, the practice of translocating “problem crocodiles” needs to be re-evaluated. Not only are they likely to return to their capture site, unless moved from one side of a movement barrier to another, but their active movements in new areas may create HCC within other communities of people. That genetically distinct subpopulations do exist, and can be identified from tissue samples, potentially allows the origins of problem animals in a particular context to be identified. This in turn could guide efforts to reduce the number of potential problem crocodiles roaming into urban centers, where the risk of HCC is considered unacceptable, by increasing intervention such as removal or harvest pressure based on new understanding of movement barriers and sources.

## Supporting information

S1 File Genotyped data of the 50 crocodiles sampled for CPA and R scripts to analyze the data. (ZIP)

S2 File ARGOS location coordinates and associated metadata for each crocodile tracked by satellites. For the attributes of the data, see the ARGOS User’s Manual at http://www.argos-system.org/manual/index.html.
(XLSX)

S3 File Ancestry coefficients for each sampled saltwater crocodile and their likely genetic origins estimated. (XLSX)

S1 Fig Histogram of the Fis values at the filtered SNPs. (TIF)

S1 Table Bootstrap F_ST_ estimates between saltwater crocodile sampling regions with 95% confidence intervals (CI). (DOCX)
