# Supplementary figures and images for: Translocation, genetic structure and homing ability confirm geographic barriers disrupt saltwater crocodile movement and dispersal
Source: PLoS One. 2019 Aug 28;14(8):e0205862. doi: 10.1371/journal.pone.0205862 (PMC6713319; doi:10.1371/journal.pone.0205862)

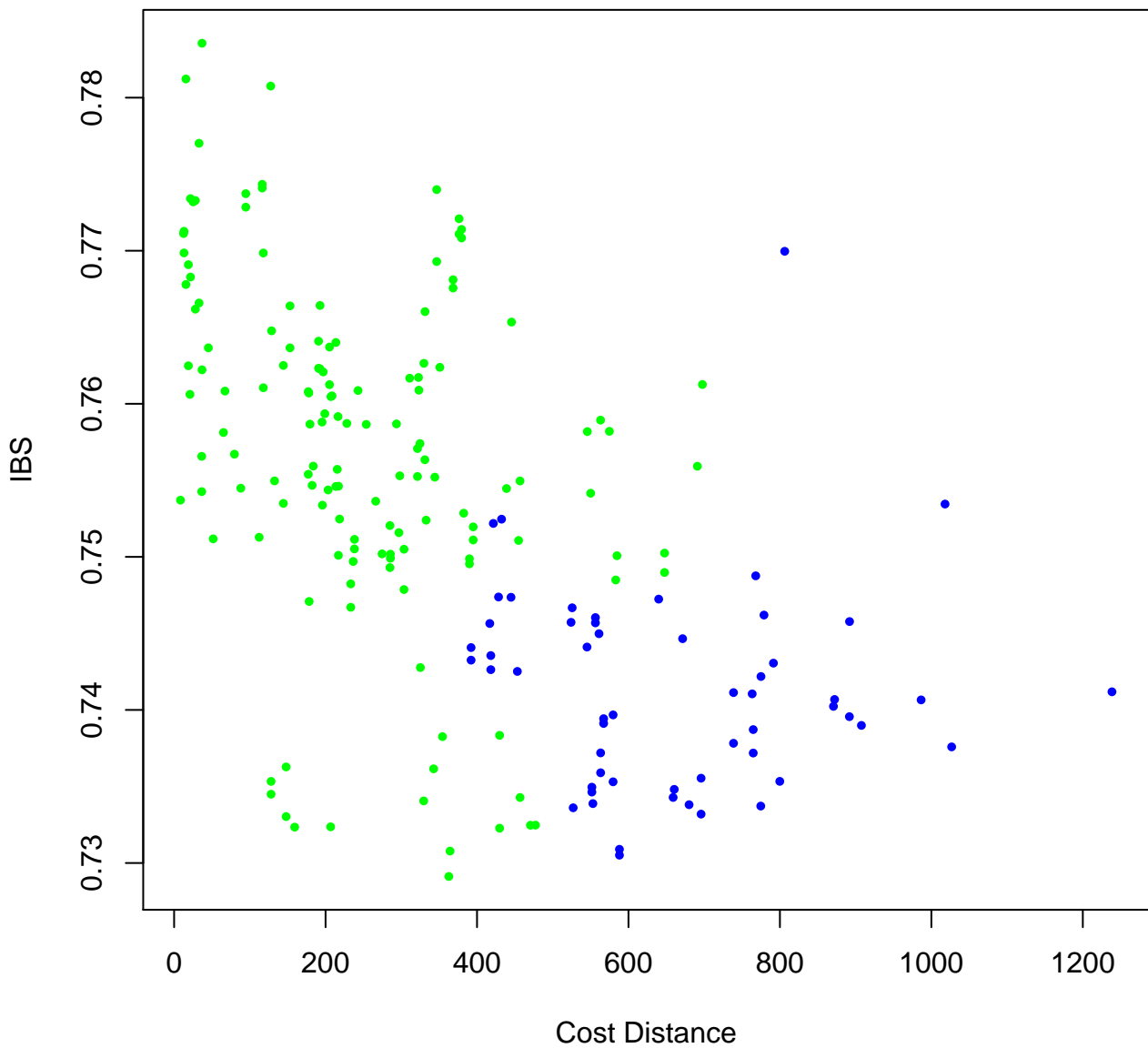

Supplement: S1 File — (ZIP) [file pone.0205862.s001.zip › Cobourg croc paper genetic analyses/IBS vs Cost Distance.pdf]

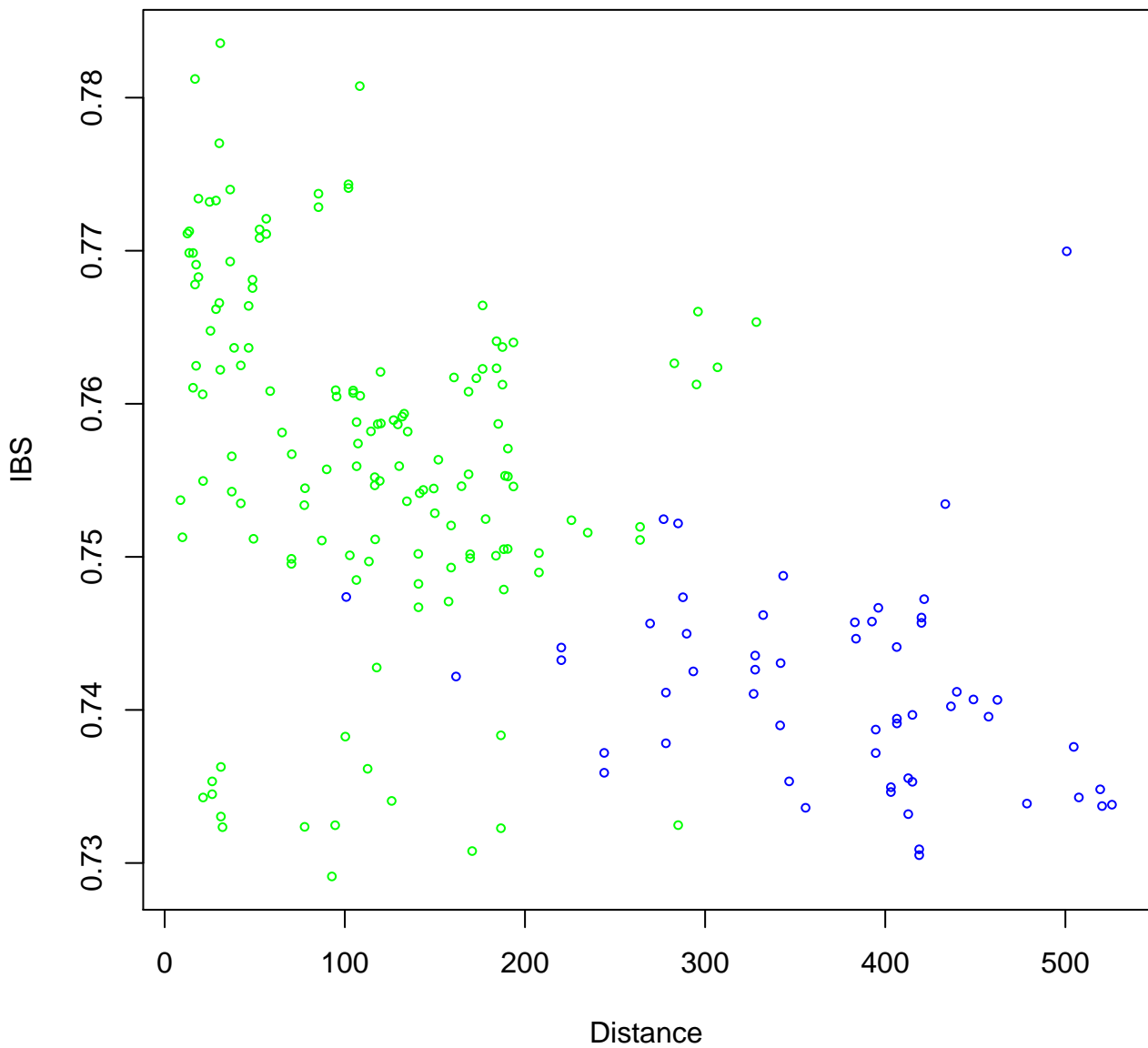

Supplement: S1 File — (ZIP) [file pone.0205862.s001.zip › Cobourg croc paper genetic analyses/IBS vs Distance.pdf]

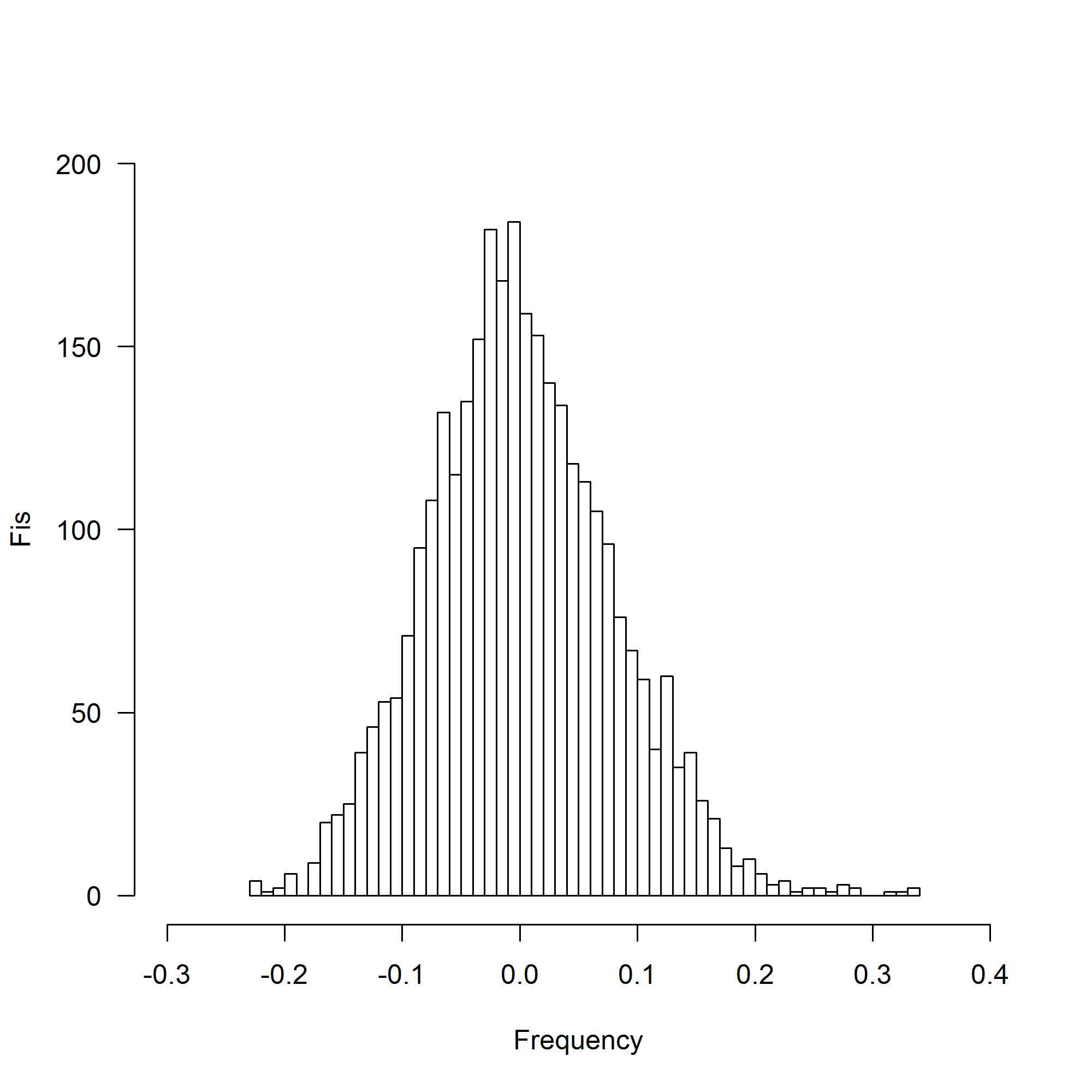

Supplement: S1 Fig — (TIF) [file pone.0205862.s004.tif]
