# Supplementary material for: Translocation, genetic structure and homing ability confirm geographic barriers disrupt saltwater crocodile movement and dispersal
Source: PLoS One. 2019 Aug 28;14(8):e0205862. doi: 10.1371/journal.pone.0205862 (PMC6713319; doi:10.1371/journal.pone.0205862)
Supplement: S1 Table — (DOCX) [file pone.0205862.s005.docx]

| **Region 1** | **Region 2** | **Bootstrap F_ST_ (95% CI)** |
| --- | --- | --- |
| **Cobourg Murganella Creek** | Adelaide River | 0.019 (0.017-0.021) |
| **Cobourg Murganella Creek** | Finniss River | 0.023 (0.021-0.025) |
| **Cobourg Murganella Creek** | Mary River | 0.018 (0.016-0.02) |
| **Cobourg Murganella Creek** | Arafura Swamp* | 0.061 (0.058-0.064) |
| **Cobourg Murganella Creek** | Maningrida King River* | 0.027 (0.024-0.028) |
| **Cobourg Murganella Creek** | Tiwi Islands | 0.012 (0.011-0.014) |
| **Adelaide River** | Finniss River | 0.038 (0.035-0.04) |
| **Adelaide River** | Mary River | 0.029 (0.026-0.031) |
| **Adelaide River** | Arafura Swamp * | 0.076 (0.073-0.079) |
| **Adelaide River** | Maningrida King River* | 0.041 (0.039-0.044) |
| **Adelaide River** | Tiwi Islands | 0.023 (0.022-0.025) |
| **Finniss River** | Mary River | 0.037 (0.034-0.04) |
| **Finniss River** | Arafura Swamp * | 0.075 (0.072-0.077) |
| **Finniss River** | Maningrida King River* | 0.044 (0.042-0.046) |
| **Finniss River** | Tiwi Islands | 0.031 (0.03-0.033) |
| **Mary River** | Arafura Swamp * | 0.069 (0.066-0.073) |
| **Mary River** | Maningrida King River* | 0.034 (0.031-0.037) |
| **Mary River** | Tiwi Islands | 0.023 (0.021-0.025) |
| **Arafura Swamp *** | Maningrida King River* | 0.015 (0.014-0.016) |
| **Arafura Swamp *** | Tiwi Islands | 0.061 (0.059-0.064) |
| **Maningrida King River*** | Tiwi Islands | 0.031 (0.03-0.033) |

*Regions on the eastern side of the Cobourg Peninsula.
